# Integrative medicine utilization among infertility patients

**DOI:** 10.1186/s12958-023-01121-6

**Published:** 2023-08-02

**Authors:** Shruti Sehgal, Ashley Dyer, Christopher Warren, Isabel Galic, Tarun Jain

**Affiliations:** 1 Institute for Public Health and Medicine, Northwestern University Feinberg School of Medicine, Chicago, IL USA; 2 Division of Reproductive Endocrinology and Infertility, Department of Obstetrics and Gynecology, Northwestern University Feinberg School of Medicine, Chicago, IL USA

**Keywords:** Infertility, Complementary medicine, Alternative medicine, IVF, Acupuncture, Yoga, Mind–body medicine

## Abstract

**Background:**

Previous research suggests that some women are using integrative and complementary holistic approaches to optimize their own health and treat infertility. We aimed to determine patterns of integrative medicine use among those seeking fertility optimization by 1) Characterizing patterns of integrative medicine use to increase fertility; 2) Identifying demographic predictors associated with such integrative medicine use; and 3) Exploring cultural and religious influences on use of integrative medicine.

**Methods:**

Cross-sectional self-reported survey data were collected from 1460 patients presenting to an academic fertility center in Chicago, Illinois. Variables were described with univariate frequencies and proportions, unadjusted bivariate comparisons were made between patient-level factors and reported integrative modality use, and multivariable logistic regression evaluated the strength of covariate-adjusted predictors of reported integrative medicine utilization.

**Results:**

80.4% of respondents reported using at least one integrative medicine modality to treat infertility (Acupuncture: 38.5%, Yoga: 27.6%, Massage: 25.8%, Meditation: 16.7%, and Herbal supplements: 18.5%). Diet therapy was the most frequently utilized modality (74.0%) followed by body therapy (45.2%), traditional alternative medicine (42.0%), mind therapy (32.1%), and senses therapy (23.0%). Any integrative medicine modality use was 4.03 times more likely among Hindu respondents compared to participants that identified as not religious (95% CI 1.2–13.7, *p* < 0.026). Significant differences in specific modality use were observed by race, religious affiliation, age, income, and insurance coverage.

**Conclusion:**

Most infertility patients in our study reported using at least one integrative medicine modality to help them conceive. Utilization was associated with age of participant, religious affiliation, annual income, and insurance coverage. Further research is needed to assess the impact of integrative medicine utilization on patient quality of life and outcomes.

## Background

As reproductive technology becomes increasingly available and accessible to individuals experiencing infertility in the United States (US) [1], previous research suggests that an increasing number of women and couples are also seeking holistic approaches to optimize their own fertility and health using integrative and complementary modalities [2, 3]. According to the National Institutes of Health, complementary medicine is defined as pairing a non-mainstream approach together with conventional medical practices [4]. The overarching goal of integrative and complementary health practices places an emphasis on treating the whole person from physical, pyscho-emotional, and spiritual perspectives rather than focusing medical treatment on treating one single organ system [4].

Previous data from the 2007 National Health Interview Survey (NHIS) indicates that 67% of women of reproductive age used complementary and holistic approaches, predominately for back pain, neck pain, and anxiety [2]. Among women who were pregnant or within the postpartum period, 20% reported using complementary and holistic approaches for pregnancy-related health conditions [2]. One study by Smith et al. (2010) articulated the prevalence of integrative and complementary modalities used by couples in a prospective cohort of eight community and academic reproductive endocrinology practices where 29% of the total population reported using an integrative and complementary modality with acupuncture (22%), herbal therapy (17%), and body work (5%) being the most utilized [3].

Over a decade later, an increased understanding of the patterns of use of integrative medicine modalities among patients seeking to treat infertility is imperative to help allopathic physicians engage with patients in clinical decision-making. This may also serve as an opportunity to explore the evidence base further and articulate how allopathic and integrative clinicians may best collaborate to provide comprehensive, patient-centered care. Our study goals were to determine patterns of integrative medicine use among those seeking fertility optimization by 1) Characterizing patterns of integrative medicine use to increase fertility; 2) Identifying demographic predictors associated with such integrative medicine use; and 3) Exploring cultural and religious influences on use of integrative medicine.

## Methods

### Study setting

A cross-sectional study was conducted at the Northwestern Center for Fertility and Reproductive Medicine, within the Division of Reproductive Endocrinology and Infertility, at Northwestern University’s Feinberg School of Medicine. The study was approved by the Institutional Review Board at Northwestern University.

### Survey instrument

The 32-question survey instrument used in this study was adapted from previous work [5]. It was then reviewed and piloted by a fertility physician, non-fertility physician, licensed clinical psychologist specializing in women’s reproductive health, and five research study staff including a survey methodologist and biostatistician. The final version of the survey consisted of questions that inquired about patient demographics, infertility history, attitudes, and beliefs towards seeking fertility care, and treatments including usage of integrative modalities to improve our understanding of patient experiences with infertility and to account for technological advancements to fertility care made in the last decade.

### Study procedures

The survey, programmed into an electronic data capture platform, REDCap, was administered to 5,000 unique participants who presented to the Northwestern Center for Fertility and Reproductive Medicine for at least one visit between June 2018 and September 2019. Email addresses of participants were obtained via the Northwestern Enterprise Data Warehouse. The electronic consent form and survey were e-mailed in September of 2019 and responses were collected over the next month. Those that did not respond received two additional follow-up emails to encourage completion.

Northwestern Center for Fertility and Reproductive Medicine has three clinical sites throughout the greater Chicago area, allowing for potential patients from different locations to obtain access to care. Of the 5,000 e-mails sent, 377 were not delivered due to incorrect e-mail addresses. Out of the remaining 4,623 surveys sent, 1,460 responses were collected from participants (32% response rate). Baseline demographic data were also obtained from the Northwestern Enterprise Data Warehouse to evaluate potential nonresponse bias between survey responders and non-responders.

### Statistical analysis

Descriptive univariate frequencies, and percentages were calculated for all available categorical data to summarize participant demographics and reported Integrative Medicine Modality (IMM) use. For categorical outcomes, Pearson’s chi-squared tests were calculated to compare unadjusted quantities, while a series of multiple logistic regression models were fit to examine factors associated with reported use of each patient-reported IMM. Logistic regression models calculated odds ratios that were statistically adjusted for the following factors: age (< 35, 35–37, 38–40, 41–42, > 42); parity (parous vs. nulliparous); race/ethnicity (White, Black, Latinx, Asian, multiple/other); in-come (< $100 K, $100–200 K, $200–400 K, > $400 K); religion (Catholic, Protestant, Jewish, nonreligious or spiritual, other Christian, Hindu, other); education (less than a bachelor’s, bachelor’s, master’s, or terminal professional degrees); and insurance coverage for fertility treatment (none, < 50%, 50%–75%,or > 75% coverage). All analyses were conducted using Stata 17 (StataCorp LLC, College Station, TX). Reported *P* values were 2-sided significance tests with conventional *p* < 0.05 thresholds employed to evaluate statistical significance.

## Results

### Demographics

As seen in Table 1, most respondents in our sample (*N* = 1460) were white (72.2%), while 10.0% identified as Asian, 7.0% as Black or African American, 5.4% as Hispanic/Latino, and 5.4% as multiple or other racial/ethnic identities. Overall, most respondents held higher education degrees (Masters: 40.5%; Professional degree: 19.1%) and had an annual household income of over $100,000 (81.2%). Majority of the respondents were catholic (37.5%), under 35 years of age (35.7%), and suggested that their physician understood their cultural background (79.4%).

**Table 1:** Demographics of participants (Integrative Medicine Modality Users compared to non-users) presenting at Chicago Metropolitan Area fertility clinic network

	**Total N (%)**	**No Use of IMM N (%)**	**Any Use of IMM N (%)**	**Acupuncture N (%)**	**Yoga N (%)**	**Massage N (%)**	**Meditation N (%)**	**Herbal supplements N (%)**	**X**^**2 (any IMM vs no IMM)**^	***p*-value**
	**1460 (100)**	**286 (19.6)**	**1 174 (80.4)**	**562 (38.5)**	**403 (27.6)**	**377 (25.8)**	**244 (16.7)**	**270 (18.5)**		
**Race or ethnicity**										
** White**	1054 (72.2)	205 (19.5)	849 (80.5)	445 (42.2)***	300 (28.5)	289 (27.4)*	169 (16.0)	188 (17.8)	0.8	0.94
** Black or AA**	102 (7.0)	23 (23.6)	79 (77.4)	29 (28.4)***	21 (20.6)	25 (24.5)*	21 (20.6)	25 (24.5)		
** Hispanic/Latino**	79 (5.4)	14 (17.7)	65 (82.3)	17 (21.5)***	19 (24.1)	20 (25.3)*	15 (19.0)	11 (13.9)		
** Asian**	146 (10)	29 (19.9)	117 (80.1)	41 (28.1)***	35 (24.0)	21 (14.4)*	23 (15.8)	24 (16.4)		
** Multiple/Other**	79 (5.4)	15 (19.0)	64 (81.0)	30 (38.0)***	28 (35.4)	22 (27.9)*	16 (20.3)	22 (27.9)		
**Relationship Status**										
** Single**	99 (6.8)	47 (47.5)	52 (52.5)***	19 (19.2)***	15 (15.2)**	17 (17.2)	12 (12.1)	6 (6.1)**	55.4	< 0.001
** Heterosexual Relationship**	1239 (85.0)	210 (17.0)	1029 (83.1)***	501 (40.4)***	363 (29.3)**	335 (27.0)	215 (17.4)	244 (19.7)**		
** Divorced or Separated**	16 (1.1)	4 (25.0)	12 (75.0)***	5 (31.3)***	5 (31.3)**	5 (31.3)	4 (25.0)	6 (37.5)**		
** Same Sex Relationship**	74 (5.1)	16 (21.6)	58 (78.4)***	32 (43.2)***	17 (23.0)**	14 (18.9)	9 (12.2)	8 (10.8)**		
** Other**	29 (2.0)	7 (24.1)	22 (75.9)***	5 (17.2)***	3 (10.3)**	6 (20.7)	4 (13.8)	6 (20.7)**		
**Religion**										
** Catholic**	531 (37.5)	95 (17.9)	436 (82.1)	226 (42.6)**	162 (30.5)	155 (29.2)*	94 (17.7)	112 (21.1)**	9.1	0.17
** Other Christian**	102 (7.2)	14 (13.7)	88 (86.3)	44 (43.1)**	33 (32.4)	34 (33.3)*	15 (14.7)	30 (29.4)**		
** Protestant**	212 (15.0)	42 (19.8)	170 (80.2)	79 (37.3)**	58 (27.4)	48 (22.6)*	35 (16.5)	34 (16.0)**		
** Judaism**	122 (8.6)	27 (22.1)	95 (77.9)	49 (40.2)**	32 (26.2)	23 (18.9)*	19 (15.6)	17 (13.9)**		
** Hinduism**	48 (3.4)	5 (10.4)	43 (89.6)	15 (31.3)**	11 (22.9)	7 (15.6)*	11 (22.9)	5 (10.4)**		
** Secular/Agnosticism/ Non-religious**	360 (25.4)	83 (23.1)	277 (76.9)	124 (34.4)**	90 (25.0)	88 (24.4)*	50 (13.9)	65 (18.1)**		
** Other**	43 (3.0)	8 (18.6)	35 (81.4)	7 (16.3)**	9 (20.9)	10 (23.3)*	8 (18.6)	1 (2.3)**		
**Education**										
** Less than Bachelor's**	76 (5.2)	19 (25.0)	57 (75.0)	11 (14.5)***	9 (11.8)**	17 (22.4)**	7 (9.2)	13 (17.1)	18.2	< 0.001
** Bachelors**	512 (35.1)	85 (16.6)	427 (83.4)	217 (42.4)***	162 (31.6)**	150 (29.3)**	92 (18.0)	103 (20.1)		
** Master’s Degree**	591 (40.5)	104 (17.6)	487 (82.4)	241 (40.8)***	168 (28.4)**	158 (26.7)**	99 (16.8)	115 (19.5)		
** Professional Degree**	279 (19.1)	78 (28.0)	201 (72.0)	93 (33.3)***	63 (22.6)**	52 (18.6)**	46 (16.5)	39 (14.0)		
**Annual Household Income**										
** < $100,000**	271 (18.8)	66 (24.4)	205 (75.6)	77 (28.4)***	53 (19.6)**	52 (19.2)*	37 (13.7)	46 (17.0)*	5	0.08
** $100,001—$200,000**	589 (40.9)	108 (18.3)	481 (81.7)	220 (37.4)***	180 (30.6)**	150 (25.5)*	103 (17.5)	130 (22.1)*		
** > $200,00**	580 (40.3)	197 (18.5)	473 (81.5)	258 (44.5)***	164 (28.3)**	168 (29.0)*	100 (17.2)	91 (15.7)*		
**Insurance Coverage for Fertility Treatment**										
** No Coverage**	264 (18.4)	62 (23.5)	202 (76.5)	87 (33.0)	62 (23.5)	61 (23.1)	40 (15.2)	41 (15.5)	5.3	0.15
** < 50% Coverage**	83 (5.8)	19 (22.9)	64 (77.1)	32 (38.6)	26 (31.3)	26 (31.3)	19 (22.9)	16 (19.3)		
** 50%-74% Coverage**	300 (20.9)	62 (20.7)	238 (79.3)	116 (38.7)	83 (27.7)	72 (24.0)	50 (16.7)	61 (20.3)		
** 75%-100% Coverage**	789 (54.9)	139 (17.6)	650 (82.4)	320 (40.6)	227 (28.8)	213 (27.0)	132 (16.7)	149 (18.9)		
**Age**										
** < 35**	515 (35.7)	113 (21.9)	402 (78.1)	167 (32.4)**	141 (27.4)	120 (23.3)	82 (15.9)	84 (16.3)*	7.6	0.11
** 35–37**	402 (27.9)	82 (20.4)	320 (79.6)	160 (39.8)**	105 (26.1)	100 (24.9)	62 (15.4)	72 (17.9)*		
** 38–40**	286 (19.8)	50 (17.5)	236 (82.5)	124 (43.4)**	74 (25.9)	81 (28.3)	49 (17.1)	55 (19.2)*		
** 41–42**	132 (9.2)	25 (18.9)	107 (81.1)	56 (42.4)**	47 (35.6)	37 (28.0)	22 (16.7)	26 (19.7)*		
** > 42**	107 (7.4)	12 (11.2)	95 (88.8)	48 (44.9)**	33 (30.8)	34 (31.8)	26 (24.3)	31 (29.0)*		
**Do you feel your fertility physician understands your cultural background?**										
** Yes**	1142 (79.4)	213 (18.8)	920 (81.2)	**436 (38.5)**	312 (27.5)	300 (26.5)	175 (15.5)*	**199 (17.6)**		
** No**	296 (20.6)	62 (21.1)	232 (78.9)	**114 (38.8)**	84 (28.6)	70 (23.8)	62 (21.1)*	**66 (22.5)**		

Statistically significant differences in the distribution of specific demographic factors among respondents reporting use of any or specific IMM modalities are indicated by asterisks (i.e. *two-sided omnibus chi-square *p *<.05; ** two-sided omnibus chi-square *p *<.01; *** two-sided omnibus chi-square *p *<.001)

### IMM use

Table 1 shows that 80.4% of respondents reported using at least one IMM to increase their chance of getting pregnant (Acupuncture: 38.5%, Yoga: 27.6%, Massage: 25.8%, Meditation: 16.7%, Herbal supplements: 18.5%). There were no significant differences in overall IMM use by racial group or religious affiliation in this sample. There was a significant difference in IMM usage by relationship status with fewer respondents that identified as “single” reporting IMM use (52.5%) compared to those in a heterosexual relationship (83.1%) (*p* < 0.0001). There was some difference in IMM usage across education levels with 83.4% of the respondents with a bachelor’s degree and 82.4% with a master’s degree suggesting IMM usage, when compared to those that suggested less than a bachelor’s degree (75.0%). A greater number of respondents with an income more than or equal to $100,000 reported using at least one IMM ($100,001—$200,000: 81.7% and > $200,000: 81.5%) than those with an income of less than $100,000 (75.6%). Frequency of IMM usage directly increased as insurance coverage increased with a 6% difference between those reporting no insurance coverage and those reporting full insurance coverage for fertility treatment (No coverage: 76.5%, 75%-100% coverage: 82.4%). There was also a 10% difference in percentage of people using any IMM between those under 35 years (78.1%) and over 42 years of age (88.8%).

### Acupuncture

Acupuncture use to assist with fertility was reported in 38.5% of all survey respondents, with significant differences by race, religious affiliation, relationship status, income, education, and age. A majority of these respondents identified as white (42.2%), or “multiple/other” racial identities (38.0%). Those in heterosexual or same sex relationships reported higher utilization of acupuncture (40.4% and 43.2% respectively) than those who reported being single or divorced or separated (19.2% and 31.3%% respectively). The highest percentage of acupuncture use (44.5%) was among respondents whose income was greater than $200,000 annually. Similarly, those whose insurance covered 75–100% of their fertility treatment more commonly reported using acupuncture (40.6%) than those with no coverage (33.0%). Acupuncture utilization was more common among those over the age of 42 (44.9%) than those younger than 35 years (32.4%).

### Yoga

27.6% of all respondents in this sample reported having practiced yoga to assist with fertility. There were significant differences by relationship status, education, and income. Yoga was least common among those that reported being single (15.2%). Most frequent yoga use was reported among those with a bachelor’s or master’s degree (31.6% and 28.4%). About one third of those that reported practicing yoga for fertility were Catholic, Protestant, or “Other Christian,” (30.5, 27.4, and 32.4% respectively). Yoga was also more common among those with an income of over $100,000 ($100,001-$200,000: 30.6% and > $200,000: 28.3%).

### Massage

About one fourth of this sample (25.8%) utilized massage for fertility, with significant differences by race, religion, education, and income. Massage was commonly used by respondents who identified as White (27.4%) and Hispanic/Latino (25.3%), followed by Black (24.5%,) and Asian (14.4%). Among religious groups, participants that used massage were primarily Catholic or other Christian (29.2% and 33.3% respectively). About a third (29.0%) of those that use massage also had an annual income of over $200,000.

### Meditation

Meditation was utilized for fertility among 16.7% of respondents in the study. Among religious groups, meditation was most common among those that identified as Hindu (22.9%). It was also slightly more common among those older than 42 years (24.3%) compared to those under 35 (15.9%).

### Herbal supplements

Almost one fifth of respondents in the sample (18.5%) took herbal supplements to assist with fertility, with significant differences by religious affiliation and relationship status. Almost a third of those that took herbal supplements were over the age of 42 years (29.0%).

**Fig. 1:**
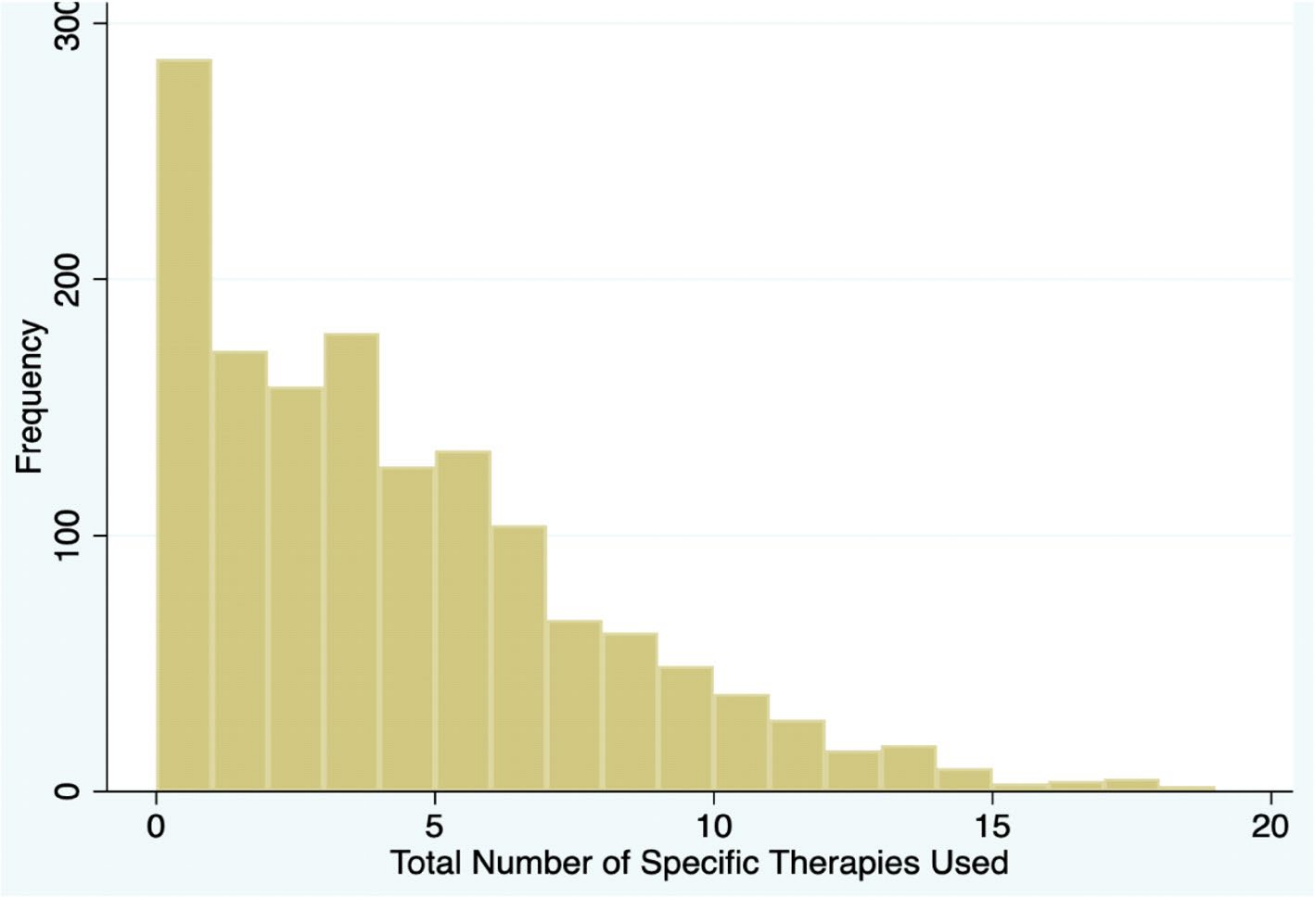
Distribution of total number of specific integrative medicine modalities (IMM) used

Table 2 describes the overall frequency of every integrative medicinal modality queried in the survey. For each modality category, participants were able to select multiple modality responses or “none of the above.” Diet therapy was the most frequently utilized modality (74.0%) followed by body therapy (45.2%), traditional alternative medicine (42.0%), mind therapy (32.1%), and senses therapy (23.0%). Among diet therapies, Vitamin D, other vitamins, and CoQ10 were most frequently utilized (43.2%, 44.7%, and 35.4% respectively). Among non-diet related modalities, acupuncture (38.5%), yoga (27.6%), massage (25.8%), and meditation (16.7%) were the most frequently reported. Furthermore, the majority of the respondents in our study used 1–5 integrative medicine modalities for fertility treatment (Fig. 1).

**Table 2:** Overall frequencies by integrative modality as reported by participants

Total *N* = 1460	**N**	**%**
**Traditional Alternative Medicine**	**613**	**42.0**
Acupuncture	526	38.5
Chinese Medicine	164	11.2
Naturopathy	97	6.6
Ayurveda	26	1.8
None of the above [response option]	847	58
**Body Therapy**	**660**	**45.2**
Yoga	403	27.6
Massage	377	25.8
Chiropractor	168	11.5
Energy field therapy	38	2.6
Tai Chi	3	0.2
None of the above [response option]	800	54.8
**Mind Therapy**	**469**	**32.1**
Meditation	244	16.7
Psychotherapy	189	13
Biofeedback	5	0.3
None of the above [response option]	991	67.9
**Senses Therapy**	**336**	**23.0**
Visualization and guided imagery	108	7.4
Music	97	6.6
Dance	41	2.8
None of the above [response option]	1124	77
**Diet Therapy**	**1,081**	**74.0**
Other vitamins	652	44.7
Vitamin D	631	43.2
CoQ10	517	35.4
Nutrition/weight loss regimens	392	26.9
DHEA	380	26
Specific fruits/veg	335	23
Herbal supplements	270	18.5
None of the above [response option]	379	26

### Factors associated with the use of integrative medicine

#### Predictors of any integrative medicine use

**Fig. 2:**
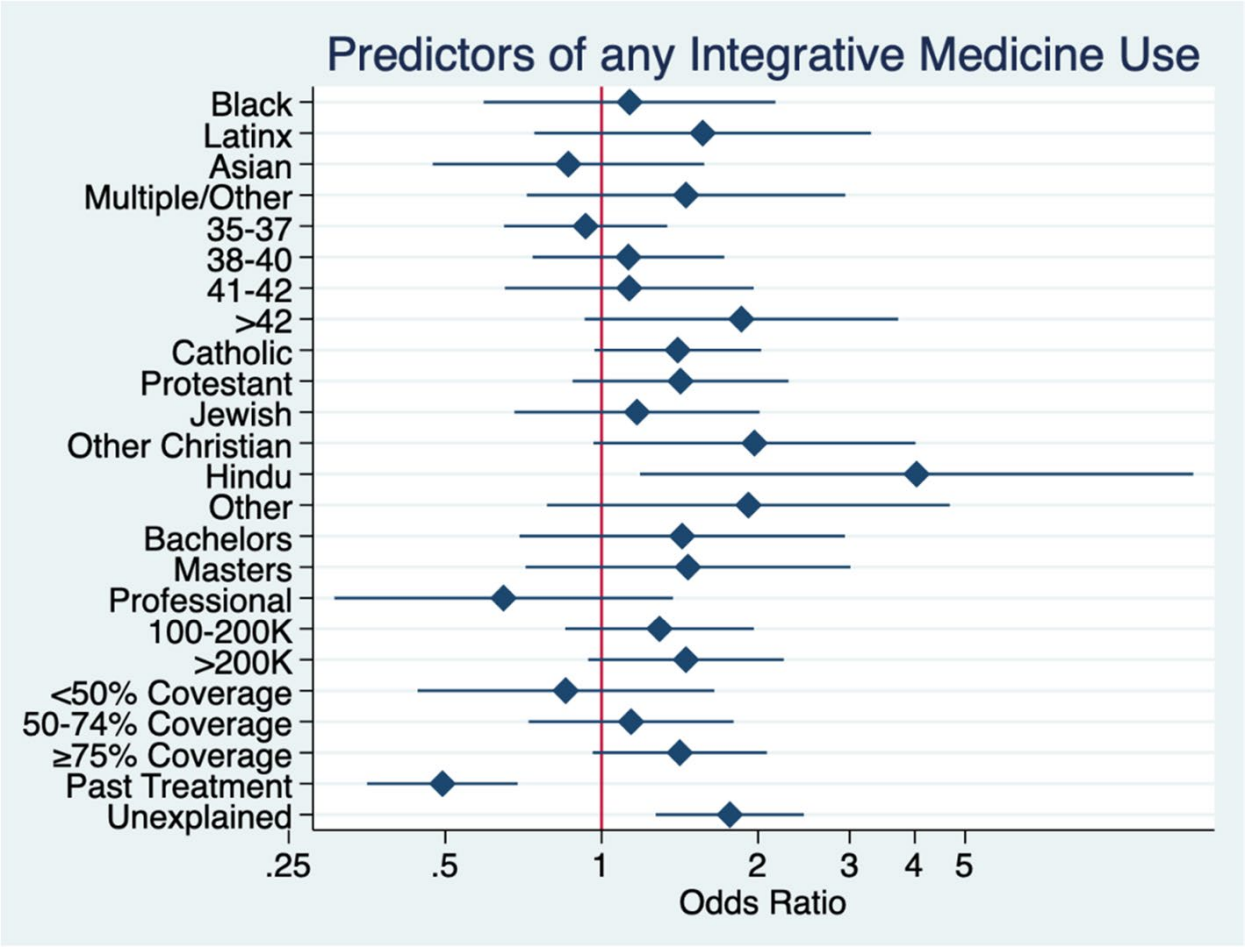
Factors associated with any integrative medicine used for fertility treatment

Illustrated in Fig. 2, any IMM use was 4.03 times more likely among Hindu respondents compared to participants that identified as not religious (95% CI 1.2–13.7, *p* < 0.026). Hispanic/Latino race (OR 1.6, 95% CI 0.8–3.3), age over 42 years (OR 1.9, 95% CI 0.9–3.7), income > $200,000 (OR 1.5, 95% CI 0.9–2.2, and higher insurance coverage (OR 1.4, 95% CI 0.96–2.1) for fertility treatment was associated with an increased likelihood of integrative medicine use. Overall IMM use was 1.8 times more likely in those with an unexplained cause of infertility (95% CI 1.3–2.5, *p* < 0.001) compared to those who knew their cause of infertility.

**Fig. 3:**
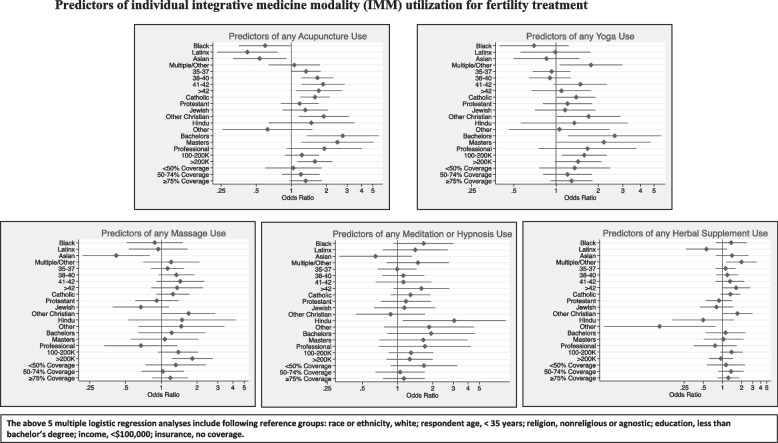
Predictors of individual integrative medicine modality (IMM) utilization for fertility treatment

Specific modalities were also analyzed by demographic predictors that describe trends in the population, i.e., by race, age, religious affiliation, education, income, and insurance status (Fig. 3).

### Predictors of acupuncture use

Black, Latinx, and Asian participants were significantly less likely than white participants to use acupuncture (OR 0.6, 95% CI 0.4–1.0; OR 0.4, 95% CI 0.2–0.8; OR 0.5, 95% CI 0.3–0.9 respectively). Compared to respondents who were under 35 years of age, those who were above 40 were more likely to use acupuncture (41–42 OR 1.9, 95% CI 1.2–2.9 and > 42 OR 1.7, 95% CI 1.1–2.7). Catholics were 1.6 times (95% CI 1.2–2.) and other Christians were 1.9 times (95% CI 1.2–3.1) more likely to report acupuncture use than their non-religious counterparts. Those with an income of greater than $200,000 were significantly more likely than those with an income of less than $100,000 to use acupuncture (OR 1.6, 95% CI 1.1–2.3, *p* < 0.008).

### Predictors of yoga use

Respondents who identified as part of “multiple” or “other” racial groups were significantly more likely to utilize yoga (OR 1.8, 95% CI 1.1–3.0) than their White counterparts. Compared to respondents who were under 35 years of age, those who were above 40 were more likely to use yoga (41–42 OR 1.5, 95% CI 1.0–2.3 and > 42 OR 1.1, 95% CI 0.7–1.8). Participants with an income greater than $100,000 were significantly more likely to use yoga than those with an income of less than $100,000 (100-200 K OR 1.6, 95% CI 1.1–2.3 and > 200 K OR 1.4, 95% CI 1.0–2.1). Catholics were 1.4 times (95% CI 1.0–1.9), other Christians were 1.7 times (95% CI 1.0–2.9), and Hindus were 1.3 times (95% CI 0.6–3.3) more likely than their non-religious counterparts to use Yoga as an integrative modality. Bachelor’s and master’s degree respondents were more than twice as likely to use yoga as their counterparts with no bachelor’s degree (OR 2.6, 95% CI 1.2- 5.6; OR 2.2, 95% 1.0–4.7 respectively).

### Predictors of massage use

While Asian participants were less likely to use massage (OR 0.42, 95% CI 0.2–0.8), those that identified as part of “multiple” or “other” racial groups were significantly more likely to use massage (OR 1.2, 95% CI 0.7–2.1) than their White counterparts. Participants over the age of 35 were significantly more likely than those under 35 to utilize massage (35–37 OR 1.1, 95% CI 0.8–1.6; 38–40 OR 1.3, 95% CI 0.9–1.9; 41–42 OR 1.4, 95% CI 0.9–2.3; > 42 OR 1.4, 95% CI 0.8–2.2). Compared to non-religious respondents, Catholics, other Christians, and Hindus were more likely to report using massage (OR 1.3, 95% CI 0.9–1.7; OR 1.7, 95% CI 1.0–2.8; OR 1.5, 95% CI 0.5–4.2 respectively).

### Predictors of meditation use

Meditation and hypnosis use was significantly higher among Black (OR 1.7, 95% CI 0.9–3.0), Latinx (OR 1.4, 95% CI 0.8–2.7), and multiple/ other race (OR 1.5, 95% CI 0.8–2.7) respondents, but lower among Asians (OR 0.7, 95% CI 0.3–1.3) when compared to their White counterparts. Other significant predictors for meditation use were age more than 42 (OR 1.6, 95% CI 0.9–2.8), Hinduism (OR 3.0, 95% CI 1.1–8.4), master’s or professional degree (OR 1.6, 95% CI 0.7–3.9; OR 1.7, 95% CI 0.7–4.2 respectively), and less than 50% insurance coverage (OR 1.7, 95% CI 0.9–3.2).

### Predictors of herbal supplements use

Respondents who identified as Black and Asian were 1.4 times more likely (95% CI 0.8–2.4 and 95% CI 0.8–2.6 respectively) to use herbal supplements than their White counterparts. Compared to those who were less than 35 years of age, respondents who were more than 42 were 1.6 times more likely to use herbal supplements (95% CI 1.0–2.8). While Catholic (OR 1.3, 95% CI 0.9–1.9) and other Christians (OR 1.7, 95% CI 1.0–3.0) had higher likelihood for herbal supplement use, Protestant (OR 0.9, 95% CI 0.6–1.4), Jewish (OR 0.8, 95% CI 0.4–1.5), and Hindu respondents (OR 0.5, 95% CI 0.2–1.5) had a significantly lower likelihood. Other significant predictors identified for herbal supplement use were bachelor’s or master’s degree (OR 1.1, 95% CI 0.5–2.2 and OR 1.0, 95% CI 0.5–2.1 respectively), and 100-200 K annual income (OR 1.4, 95% CI 0.9–2.1).

## Discussion

To our knowledge this is the first published study to analyze predictors of IMM use by each modality (ranging from traditional alternative medicine to diet therapy) within a population of fertility patients in the US. Our study found that at least one IMM was used as an adjunct to fertility care by 80.4% of the respondents. Acupuncture, yoga, and massage were the most utilized IMM (38.5%, 27.6%, and 25.8%, respectively) while a smaller fraction of study participants utilized herbal supplements and meditation (18.5% and 16.7% respectively). Most of the respondents reported using 1–5 modalities.

Compared to prior studies on the use of integrative methods, our study shows an increase in IMM use in the last decade. Using data from the NHIS, Clarke et al. reported that complementary health approach use among U.S. adults was 34% in 2012 [6]. Similar to NHIS prevalence data between 2002 and 2012 [6, 7], Scott et al. observed increases in the use of meditation, chiropractic, and massage therapy [8]. Yoga was the most commonly used complementary health approach among U.S. adults in 2012 (9.5%) and 2017 (14.3%) [7]. This trend is consistent with our findings that show that 27.6% of all participants in our sample reported having practiced yoga to assist with fertility. Significant differences were observed by relationship status, religious affiliation, and income. Yoga was least common among those that reported being single (15.2%). About one third of those that reported practicing yoga for fertility were Catholic, Protestant, or “Other Christian,” (30.5, 27.4, and 32.4% respectively). It was also more common among those with an income of over $100,000 (100 K-200 K: 30.6% and > 200 K: 28.3%).

Furthermore, IMM utilization in our study is much higher than an 18-month prospective cohort study in Northern California that reported a 29% complementary and alternative medicine (CAM) use among couples seeking fertility treatment [3]. Our study also indicates that specific methods such as acupuncture use may have nearly doubled in the last decade, with 22% of couples seeking fertility care reporting acupuncture use in 2010 [3] compared to nearly 39% of participants in our study. Similarly, meditation practice appears to be more commonly practiced with 1% reporting meditation use in 2010 [3] compared to nearly 17% of participants in 2019. Although many traditional alternative and dietary modalities queried in our study originate from the healing traditions of non-Western countries where they are well-accepted and regarded as primary healing practices, a greater number of patients are turning to complementary and alternative therapies, even in US, for several reasons. First reason is due to a recent shift in US healthcare towards a more holistic and individualized approach to healing, resulting in an increasing demand [9, 10]. Second reason may be due to the increased availability of complementary medicine at a comparatively lower cost when compared to assisted reproductive technologies [11]. The third reason may be due to concern about the adverse effects of conventional medicine versus the benefit of an individualistic approach to patient care that incorporates the mind, spirituality, as well as the body [3, 11]. A recent retrospective study in Texas showed that adult hospitalized patients who received integrative medicine were more likely to have reduced pain, lowering the hospital cost by nearly 4% [12].

Another interesting observation in our study was the utilization of diet therapy by nearly three fourths of our sample. Diet is considered an effective intervention for improving fertility and reproductive health. Specifically, caloric intake and diet constituents play a significant role in the treatment of infertility caused by endometriosis and ovulation disorders. In our study, among diet therapies, Vitamin D, CoQ10, and other vitamins were the most frequently utilized (43.2%, 35.4%, and 44.7% respectively). It has been reported that integrating the diet with some nutritional supplements and antioxidants (Lipoic acid, Vitamin E, Vitamin C and CoQ10), can help improve fertility by reversing the bodily imbalance between the anti-oxidant protection and free radical (ROS) release [13, 14]. Notably, several reports suggest that majority of infertility cases due to ovulation disorders may be preventable through modifications of diet and lifestyle, particularly by increased consumption of either proteins or low-glycemic index carbohydrates [14–16]. A systematic review by Shang et al. suggests that positive dietary changes improve fertility outcomes in women with polycystic ovary syndrome (PCOS) [17], one of the common causes of female infertility, affecting 6–12% of US women of reproductive age [18]. They reported that the clinical pregnancy rate increased, especially with the Mediterranean and low-carbohydrate diets, and was shown to further improve with a longer diet treatment. Decreasing carbohydrate load can reduce circulating insulin levels, improve hormonal imbalance, and resume ovulation to improve pregnancy rates compared to the usual diet. Chavarro et al. found that higher adherence to a “fertility diet” (consisting of high consumption of monounsaturated fat, vegetable protein, high-fat dairy, low-glycemic carbohydrates, multivitamins, and iron from plants and supplements) was associated with a lower risk of ovulatory infertility [19]. Similarly, in a nested case–control study in Spain, women with the highest adherence to the Mediterranean diet (characterized by high amounts of legumes, vegetables, fruits, olive oil, unrefined cereals, moderate to high consumption of fish, wine, and low intake of meat) had lower likelihood of experiencing difficulties getting pregnant [20].

A key finding of our study was the utilization of mind therapy by 32% of the respondents. Growing evidence suggests that mind–body interventions, such as meditation, relaxation techniques, and mindfulness, can help in relieving the stress and depression experienced by infertile patients, thereby improving their quality of life [21, 22]. Further, mind/body therapy is shown to increase pregnancy rates and provide patients with skills in cognitive behavior therapy, relaxation training, lifestyle changes, self-awareness, and social support components [23]. Li et al. demonstrated the effectiveness of a mindfulness-based intervention among women undergoing their first IVF treatment [24]. A significant increase in mindfulness, self-compassion, meaning-based coping strategies, and higher pregnancy rates were observed in the intervention arm [24]. Rooney et al. have described how mindfulness is commonly used as a coping strategy for infertility patients and is introduced early in the therapy [25]. Keeping in mind these potential benefits, it will be necessary to develop culture-specific mind–body intervention programs for infertile women and conduct randomized control trials to examine the impact of these programs on their anxiety, depression, quality of life, and live-birth rates.

Similar to our study, Smith et al. also found overall IMM use to be directly related to female age and higher income. Bivariate analyses in their study demonstrated that each five-year increase in a woman’s age was associated with a 28% increase in the odds of CAM use (OR 1.28, *p* = 0.03). In our study, women aged over 42 years were nearly twice (OR 1.9, 95% CI 0.9–3.7) as likely to use integrative medicine when compared to those under 35 years. Further, they reported that couples with an annual household income greater than $200,000 were twice as likely to use integrative methods than those earning less than $100,000. This is in line with our study findings showing that participants with an income of greater than $200,000 were nearly 1.5 times more likely to use integrative methods than those with an income of less than $100,000.

Our study also considered insurance status and religious affiliation, which no other studies describing integrative method use among fertility population have previously assessed. Frequency of IMM usage directly increased as insurance coverage increased with a 6% difference between those reporting no insurance coverage and those reporting full insurance coverage for fertility treatment. Compared to no coverage, higher insurance coverage (greater than or equal to 75%) for fertility treatment was associated with an increased likelihood of integrative medicine use (OR 1.4, 95% CI 0.96–2.1). Any IMM use was 4 times more likely among Hindu respondents (OR 4.03, 95% CI 1.2–13) and twice among Other Christians (95% CI 1.0–4.0) compared to participants that identified as not religious. A recent work by Ellison et al. also demonstrates that spirituality and religiousness are independent predictors of certain types of CAM use. Compared to spiritual only persons in their study, spiritual and religious individuals were 43% more likely to use body-mind therapies in general and religious only individuals were disinclined toward CAM use [26]. As seen in prior work in Canada, an individual’s tradition and culture might play a role in their attitude towards integrative medicine use [27]. We observed that compared to non-religious respondents, those who identified themselves as Catholics and other Christians were nearly twice as likely to utilize acupuncture, yoga, massage, and herbal supplements. While Catholic and other Christians had higher likelihood for herbal supplement use, Protestant, Jewish, and Hindu respondents had a significantly lower likelihood. Hindus had the highest likelihood of meditation use as an integrative treatment modality.

Among racial groups, Black participants were significantly less likely than white participants to use acupuncture, but more likely to use meditation, and herbal supplements. Meditation utilization was also higher among Hispanic/Latinx. While Asians were less likely to use acupuncture, meditation, and massage, they were more likely to use herbal supplements. Respondents who identified as part of “multiple” or “other” racial groups were significantly more likely to utilize yoga. Further, higher education level and insurance coverage were both associated with an increased likelihood of utilization for all modalities examined in our study.

Further research from other geographic regions is needed to advance our understanding of factors that drive patients to seek alternative medical treatments for infertility. There is a discernible need to acknowledge the traits and motivations of infertility patients who utilize integrative medicine to augment fertility, as well as the essential influences and informational sources that influence their choices. Incorporating integrative medicine modalities within the context of a patient’s medical management may improve their quality of life and lead to better patient outcomes.

## Strengths and limitations

A strength of our study was the size of our sample, which was large, demographically similar, and geographically representative of the population of those being studied. Importantly however, this population does not include individuals who experience infertility but do not have access to fertility care. It is likely that integrative modalities are more widely and commonly utilized by those not seeking fertility support in a Western clinical setting. Also, the prevalence of IMM use in this study may have been over- or under-reported because of recall bias on the part of respondents. Lastly, the relatively small number of non-white respondents in our study limits our ability to adequately characterize use of IMM for fertility purposes in this important population. Further research that includes a more racially, ethnically, geographically, and socioeconomically diverse sample of individuals experiencing infertility may reveal a more comprehensive understanding of the role of integrative medicine modalities in the experience of infertility.

## Conclusion

In summary, 80.4% of infertility patients utilized at least one IMM to improve their chance of getting pregnant. Acupuncture, yoga, and massage were the commonly utilized modalities, while a smaller fraction utilized herbal supplements and meditation. IMM use was associated with age of participant, religious affiliation, annual income, and insurance coverage. Further research is needed to assess the motivations and impact of IMM use on patient quality of life and outcomes.

## Data Availability

All data analyzed during this study are included in this published article. The datasets used during the current study are available from the corresponding author on reasonable request.

## Abbreviations

CAM: Complementary and alternative medicine
IMM: Integrative Medicine Modality
NHIS: National Health Interview Survey
ROS: Free radical release
US: United States
